# Why Patients with Chronic Disease Keep Silent? Analysis of Item Nonresponse in Rural China

**Published:** 2019-04

**Authors:** Yiqing MAO, Zhanchun FENG, Shangfeng TANG, Tailai WU, Ruoxi WANG, Da FENG, Xiaoyu CHEN

**Affiliations:** 1. School of Medicine and Health Management, Tongji Medical College, Huazhong University of Science and Technology, Wuhan, China; 2. School of Pharmacy, Tongji Medical College, Huazhong University of Science and Technology, Wuhan, China

**Keywords:** Item nonresponse, Silence, Patients with chronic disease, Rural population

## Abstract

**Background:**

This study aimed to identify the characteristics of item nonresponse and examine the factors affecting the refusal or failure to respond of patients with chronic disease in rural China.

**Methods:**

A cross-sectional survey data from patients with chronic disease from rural China were analyzed. A total of 1,099 patients were enrolled. Chi-square test and cumulative logistic regression determined the predictors of having item nonresponse.

**Results:**

The respondents in central provinces (OR = 2.311, 95%CI = 0.532∼1.144, *P* < 0.001) with over eight household members (OR = 0.067, 95%CI = −1.632∼−0.349, *P* = 0.002), multiple chronic diseases (OR = 0.301, 95%CI = −1.673∼−0.727, *P* < 0.001), and low health knowledge level (OR = 2.112, 95%CI = 0.405∼1.090, *P* < 0.001) had more item nonresponse numbers. Compared with the participants with high school education level and above, the item nonresponse number seemed to increase when the participants were illiterate (OR = 2.159, 95%CI = 0.254∼1.285, *P* = 0.003), had primary school education (OR = 2.161, 95%CI = 0.249∼1.294, *P* = 0.004) and junior school education (OR = 2.070, 95%CI = 0.160∼1.296, *P* = 0.012).

**Conclusion:**

This study indicates the influencing factors of the item nonresponse in survey of patients with chronic disease in rural China. This study contributes to investigation practice and highlights that health institutions should improve the quality of follow-up services. Moreover, the government should pay more attention to the care of vulnerable groups, especially patients with chronic disease in rural areas.

## Introduction

Item nonresponse is defined as the sample population interviewed who did not answer some items or answered with “do not know” or “don’t have idea”. Item nonresponse is common in social surveys (1). The driving factors of item non-response include questionnaire design, data collection methods, interviewees, and respondents (2, 3). From a social science perspective, respondents have the right to keep silent on any item in a social survey, and silence leads to item nonresponse in questionnaires. The analysis of item nonresponse has theoretical, practical, and social relevance. First, in a relatively short period, the occurrence mechanisms of item nonresponse should be stably and regularly directed at a specific population. Second, the factors of item nonresponse are important evidence for designing survey programs and training interviewers and for the statistical disposition of missing data (4). Third, a survey is a social interaction process that requires interviewees to establish confidence and answer the questions of interviewers. Thus, it is more than a simple process of data collection. Analyzing item nonresponse under different social backgrounds and cultures can explore the causes of respondent silence (5).

The characteristic factors of sample populations are important influencing factors of item nonresponse. For example, when the sample is the general population, elderly participants are more willing to participate in the survey because of their stronger social conscience than younger interviewees who are more likely to refuse survey (6, 7). When the sample population has health problems, elderly respondents have less patience or energy to complete the survey (8). Individuals with high education levels easily understand the sense of the survey and participate better (9). Females have higher item nonresponse rates on worse physiology, psychology qualities, and cognitive function at a survey environment that is male dominated (10). Various factors exist, such as ethnicity, religion, income from occupation, and residential distance, in accordance with different backgrounds and samples (11, 12).

Chronic diseases are becoming increasingly serious all over the world. They now account for an estimated 80% of deaths and 70% of disability-adjusted life years lost in China (13). Patients with chronic disease have reached nearly 300 million (14). The situation in rural areas is worse. For example, the prevalence rate of diabetes in rural China increased by 6.6% from 2005 to 2013, which was much faster than that in urban areas (15). Therefore, increasing attention has been given to chronic disease research, and numerous surveys have been carried out. Patients with chronic diseases have poorer health than the non-chronic disease population. Meanwhile, patients with chronic disease have remarkable population characteristics; for example, most of them are elderly and obese and have unhealthy habits (16, 17). Characteristic factors can influence item nonresponse in a survey. However, the characteristics and influencing factors of item nonresponse in chronic disease patient surveys have not been systematically studied.

Therefore, the present study aimed to identify the characteristics of item nonresponse and find the factors affecting the silence of patients with chronic disease in rural China. Scientific evidence can guide the investigation and research of chronic diseases in the future.

## Methods

### Study Design and Sample

A cross-sectional survey study using stratified multistage sampling was conducted in April 2014. Details of the sampling method are similar with those in our previous study (18). Briefly, participants in rural areas from Henan, Chongqing, Qinghai, and Zhejiang were randomly selected to ensure sample representation. A total of 1,099 patients who had registered for resident health records participated in the survey. Questionnaires were verified through expert consultation. The interviewer team constituted of PhD and master students who were trained before the survey and required to carry out face-to-face and one-on-one interviews. Each questionnaire was completed in 30 minutes. Every participant provided a written informed consent.

The approval for this study was obtained from the Ethics Committee of Tongji Medical College, Huazhong University of Science and Technology (IORG No: IORG0003571).

### Questionnaire Design

The questionnaire used was self-designed and based on the widely available literature.

Four types of items were included: 14 characteristic, 9 health knowledge, 32 health behavior, and 8 health attitude items. 1) The characteristic items included socio-demographic characteristics, geographic accessibility, and health status. The socio-demographic characteristic items included province, age, gender, education level, occupation, etc. The geographic accessibility item was the distance from home to the nearest health institution. The health status items included kinds and age of chronic diseases. 2) The health knowledge and behavior items were developed by referring to the *2008 Chinese Citizens’ Health Literacy Survey*. The health knowledge items included the danger of passive smoking, relationship of salt and hypertension, relationship of obesity and diabetes, etc. The health behavior items included medicine intake, dietary habit, and physical examination. 3) The health attitude items included personal attitude toward follow-up services, health communication, etc.

### Data Measures

The dependent variable was the questionnaire item nonresponse. When respondents answered “I do not know,” the items were considered item nonresponse except health knowledge items. When respondents refused to answer or answered “I don’t have idea” on an item, the item was considered item nonresponse. Item nonresponse was defined by the nonresponse per questionnaire as a unit, that is, the number of item nonresponse in one questionnaire. The item non-response situation was divided into three groups (nonresponse numbers of “0”, “1–3,” and “≥ 4”) in terms of the average number.

As formulated by previous studies, socio-demographic characteristics, geographic accessibility, health status, and health knowledge level are important factors associated with item nonresponse. Hence, they were also included in the analysis of factors that influence item nonresponse. The 11 independent variables were socio-demographic characteristics that included 1) province; 2) age; 3) gender; 4) education level; 5) occupation; 6) number of household members; 7) household income; 8) distance from home to the nearest health institution; 9) kinds and 10) age of chronic diseases; and 11) health knowledge level. The latter was measured on the basis of nine closed-ended questions related to chronic diseases and divided into low, middle, and high groups in terms of the average scores.

### Statistical Analysis

All data were independently double-entered and validated using EpiData. All statistical analyses were performed using SPSS (Chicago, IL, USA). All variables were presented as frequency distribution and percentage. Chi-square test was used to examine the associations of the questionnaire’s item nonresponse with independent variables. Only variables with statistically significant differences were included in the cumulative logistic regression model. Values with *P*<0.05 (two tailed) were considered statistically significant.

## Results

### Sample Characteristics

The majority of the participants came from western provinces (58.1%), and were more than 60 years old (73.4%). Almost half of the participants were illiterates (47.5%), and had five to seven members in their households (44.6%). Most of the respondents (93.7%) only had one kind of chronic disease, and half of the patients (54.6%) had 3 to 8 years of illness age. Less than half of the participants (44.8%) showed middle health knowledge level, and high health knowledge level only accounted for 18.8% (Table 1).

**Table 1:** Socio-demographic characteristics, geographic accessibility, health status, and health knowledge level of Chinese rural chronic disease patients

***Characteristics***		***Frequency n=1099***	***Percentage %***
Province			
	Eastern	244	22.2
	Central	217	19.7
	Western	638	58.1
Age (yr)			
	≤44	37	3.4
	45–59	256	23.3
	60–74	566	51.5
	75–89	224	20.4
	≥90	16	1.5
Gender			
	Male	495	45.0
	Female	604	55.0
Education Level			
	Illiteracy	522	47.5
	Primacy School	359	32.7
	Junior School	143	13.0
	Above the High School	75	6.8
Occupation			
	Inoccupation	163	14.8
	Famers	836	76.1
	Herders	17	1.5
	Businesses	48	4.4
	Migrant Worker	35	3.2
Number of Household Members			
	Live Alone	71	6.5
	2–4	460	41.9
	5–7	490	44.6
	≥8	78	7.1
Household income			
	≤10,000	368	33.5
	10,001–20,000	297	27.0
	>20,000	434	39.5
Distances from home to the nearest health institution			
	<1km	867	78.9
	1–2km	152	13.8
	>2km	80	7.3
Multiple Chronic Diseases			
	Yes	69	6.3
	No	1030	93.7
Age of Chronic Diseases			
	≤2	191	17.4
	3–8	600	54.6
	≥9	308	28.0
Health Knowledge Level			
	Low	400	36.4
	Middle	492	44.8
	High	207	18.8

### Item Nonresponse Situation of Chronic Disease Patient Survey

The questionnaire included 14 characteristics items (22.2%), 9 health knowledge items (14.3%), 8 health attitude items (12.7%), and 32 health behavior items (50.8%). Among the four types of items, the household income item (21.8%) had a high item nonresponse rate. Of the rural residents, 14.9% did not respond to the danger of passive smoking in health knowledge items. Participants had a high nonresponse rate on the follow-up services of health attitude items on doctor’s skill (20.2%), doctor’s attitude (19.1%), and healthcare environment (19.5%). Finally, follow-up services time (11.1%) accounted for the highest nonresponse rate among the health behavior items. The item nonresponse was measured by one questionnaire. A “0/1–3/≥4” item nonresponse indicates that the item nonresponse number was zero/one to three/equal or above four in one questionnaire.

Among the 1,099 questionnaires, the questionnaire numbers of “0/1–3/≥4” item nonresponse were 462/465/172, accounting for 42.0%/42.3%/15.7% of all questionnaires.

### Predictors Affecting Item Nonresponse Situation of Chronic Disease Patient Survey

Significant differences were found between the item nonresponse numbers of the 0, 1–3, and ≥ 4 groups on various variables. The participants with more item nonresponse numbers seemed to be in the central provinces of rural China; have low education level, no occupation, and over eight household members; and live over 2 km from healthcare facilities. They were more likely to suffer from multiple chronic diseases, long illness age, and low health knowledge level (Table 2). Cumulative logistic regression analysis was then performed to examine the potential predictors of item nonresponse. Five variables, namely, province, education level, household size, kinds of chronic diseases, and health knowledge level, were finally retained in the cumulative logistic regression model (Table 3).

**Table 2:** Correlations between the item nonresponse and demographics characteristics, geographic accessibility, health status, and health knowledge level of Chinese rural chronic disease patients

***Characteristics***		***Item Nonresponse Numbers***						***X^2^***	***P***
		***0***		***1–3***		***≥4***			
		***N***	***%***	***N***	***%***	***N***	***%***		
Province								41.418	0.000
	Eastern	99	21.40	110	23.70	35	20.30		
	Central	53	11.50	124	26.70	40	23.30		
	Western	310	67.10	231	49.70	97	56.40		
Age (yr)								6.464	0.595
	≤44	17	3.70	16	3.40	4	2.30		
	45–59	112	24.20	110	23.70	34	19.80		
	60–74	241	52.20	235	50.50	90	52.30		
	75–89	88	19.00	97	20.90	39	22.70		
	≥90	4	9.00	7	1.50	5	2.90		
Gender								0.746	0.689
	Male	209	45.2	204	43.9	82	47.7		
	Female	253	54.8	261	56.1	90	52.3		
Education Level								14.297	0.026
	Illiteracy	203	43.9	240	51.6	79	45.9		
	Primacy School	150	32.5	148	31.8	61	35.5		
	Junior School	64	13.9	55	11.8	24	14.0		
	Above the High School	45	9.7	22	4.7	8	4.7		
Occupation								18.451	0.018
	Inoccupation	51	11.0	81	17.4	31	18.0		
	Famers	369	79.9	349	75.1	118	68.6		
	Herders	7	1.5	6	1.3	4	2.3		
	Businesses	20	4.3	14	3.0	14	8.1		
	Migrant Worker	15	11.0	15	3.2	5	2.9		
Number of Household Members								17.619	0.007
	Live Alone	41	8.9	25	5.4	5	2.9		
	2–4	174	37.7	200	43.0	86	50.0		
	5–7	220	47.6	204	43.9	66	38.4		
	≥8	27	5.8	36	7.7	15	8.7		
Household income								1.934	0.748
	≤10,000	159	34.4	156	33.5	53	30.8		
	10,001–20,000	116	25.1	130	28.0	51	29.7		
	>20,000	187	40.5	179	38.5	68	39.5		
Distances from home to the nearest health institution								13.961	0.007
	<1km	374	81.0	364	78.3	129	75.0		
	1–2km	48	10.4	78	16.8	26	15.1		
	>2km	40	8.7	23	4.9	17	9.9		
Multiple Chronic Diseases								22.665	0.000
	Yes	13	2.8	34	7.3	22	12.8		
	No	449	97.2	431	92.7	150	87.2		
Age of Chronic Diseases								9.084	0.031
	≤2	95	20.6	68	14.6	28	16.3		
	3–8	245	53.0	269	57.8	86	50.0		
	≥9	122	26.4	128	27.5	58	33.7		
Health Knowledge Level								31.103	0.000
	Low	129	27.9	185	39.8	86	50.0		
	Middle	231	50.0	197	42.4	64	37.2		
	High	102	22.1	83	17.8	22	12.8		

**Table 3:** Outcome of a cumulative logistic regression model examining predictors correlated with item nonresponse

***Characteristics***		***Reference***	***B***	***P***	***OR***	***95%CI***	
						***Lower***	***Upper***
Province							
	Central	Western	0.838	0.000	2.311	0.532	1.144
Education Level							
	Illiteracy	Above the High School	0.770	0.003	2.159	0.254	1.285
	Primacy School	Above the High School	0.771	0.004	2.161	0.249	1.294
	Junior School	Above the High School	0.728	0.012	2.070	0.160	1.296
Number of Household Members							
	Live Alone	≥8	−0.990	0.002	0.067	−1.632	−0.349
Multiple Chronic Diseases							
	No	Yes	−1.200	0.000	0.301	−1.673	−0.727
Health Knowledge Level							
	Low	High	0.748	0.000	2.112	0.405	1.090

Among all the significant predictors, the odds of rural Chinese who came from central provinces and had more item nonresponse numbers was 2.311 times greater than those who came from western provinces (OR = 2.311, 95%CI = 0.532∼1.144, *P* < 0.001). The item nonresponse numbers of participants who were illiterate (OR = 2.159, 95%CI = 0.254∼1.285, *P* = 0.003) and had primary school (OR = 2.161, 95%CI = 0.249∼1.294, *P* = 0.004) and junior school (OR = 2.070, 95%CI = 0.160∼1.296, *P* = 0.012) education levels were higher than those with high school and higher education levels. Participants living with over eight household members had more item nonresponse numbers than those who lived alone (OR = 0.067, 95%CI = −1.632∼−0.349, *P* = 0.002). Otherwise, patients with multiple chronic diseases had more item nonresponse numbers than those with single chronic disease (OR = 0.301, 95%CI = −1.673∼−0.727, *P*<0.001). As expected, the respondents who had low health knowledge levels had more item non-response numbers (OR = 2.112, 95%CI = 0.405∼1.090, *P*<0.001).

## Discussion

Through an analysis of the item nonresponse of surveys among patients with chronic disease in rural China, this study shows that the respondents in central provinces with over eight household members, have multiple chronic diseases, and have low health knowledge levels had more item nonresponse numbers. In other words, we found that silence factors of patients with chronic diseases in survey.

In terms of item types, the danger of passive smoking item had the highest nonresponse rate among the health knowledge items. This silence reflects the insufficiency of the health education program. Among the health attitude items, the item with the highest nonresponse rate was follow-up services, including the evaluation of doctor’s skill, doctor’s attitude, and health-care environment. This finding may be explained as follows. First, the respondents could not appraise if they did not receive any follow-up services. Second, they received services but were discontent with them. Therefore, they were reluctant to make an appraisal and remained silent because of reputation. Regardless of the real reason, patients are unsatisfied with the follow-up services in rural areas. A large gap among physician capacities, service environment, and management system exists in practice and requires the attention of the government. Follow-up services time has the highest nonresponse rates among the health behavior items. On the one hand, respondents may not accept service. On the other hand, the precision of answers decreases with the increase in age due to age-related declines in memory (10), especially toward the utilization of health care.

Of the 1,099 questionnaires, most of the item nonresponse numbers are under three in one questionnaire. However, the item nonresponse problem remains. This study shows that the elderly accounted for the majority of chronic disease patients. The item nonresponse increased with the cognitive function decline in the elderly (11). The high item nonresponse numbers of the elderly might be attributed to their children who may instill in them to not believe strangers in the case of sharing important personal information (10). These factors could result in patient silence. Hence, interviewers should acquire the trust of respondents, including their family members who can help in the patients’ survey.

The results of the cumulative logistic regression model revealed that several factors influence the item nonresponse numbers. First, patients who came from central provinces had more item non-response numbers than those who came from western provinces. A positive relationship exists between the program’s difficulty degree and population density (6). The population density of central provinces is generally higher than that of western provinces. Thus, central provinces have larger item nonresponse numbers in the survey than western provinces.

Second, education level is regarded as a core component in item nonresponse. Compared with the participants with high school and higher education levels, participants with education levels below high school had more item nonresponse numbers in the survey. Previous studies indicated that individuals with low education levels would remain silent because of insufficient capability to understand (19), which is common in the current study. Therefore, more information is needed to support them in the survey (20).

Third, although many respondents lived with many family members in the rural areas, many still lived alone. The respondents who lived with more than eight family members were more likely to have more nonresponse items. Uhrig’s conclusion (21) revealed a positive relationship between household size and the chance of refusal to answer. For extended families, members would put effort into the relationship with other families and may have perfunctory or silence attitude toward the survey. However, social surveys seem to be a platform that provides individuals who live alone a chance to communicate. Possibly, the low number of nonresponse items may be attributed to their feeling of loneliness and eagerness to express their idea or just talk to others. This explanation is perceived from the logic of social care. Vulnerable groups, such as patients with chronic disease or elderlies who live alone, should be paid close attention by the entire society.

Fourth, the participants with multiple chronic diseases had more item nonresponse numbers than those with one kind of disease. The time of insisting the interview influenced the rate of response (22). Patients with multiple chronic diseases have more severe health status than those with a single disease. Accordingly, the former might not have enough patience or energy toward the interview and thus remain silent in the survey. Future surveys must consider how to control interview time and be aware of the visiting environment and interviewees’ attitude.

Finally, this survey proved that rural patients have low health knowledge level. Such individuals had more item nonresponse numbers in the survey. The possible explanation is that health knowledge level represents the attention degree of caring for the self-health status. When someone pays more attention to self-health, health knowledge from all kinds of channels may be acquired. Therefore, they will actively participate in health surveys and have few item nonresponse numbers. This finding corresponds with the “knowledge–attitude–practice” model (23, 24). These results can also be used to guide the development of health education for patients with chronic disease in rural areas. Increasing the health knowledge level by all kinds of channels is beneficial to improving patient health attention, thereby achieving chronic self-management.

### Limitations

This study had several limitations. First, the variables for item nonresponse in the questionnaire are incomplete because the objective of this survey was mainly to investigate the health knowledge and behavior of patients with chronic diseases and their health service utilization. Second, these samples might not represent the conditions of all patients with chronic disease because the survey only included patients with chronic hypertension and/or diabetes, leaving other chronic diseases unstudied. Third, the factors of item nonresponse are complicated and non-control factors that could influence results of the survey remain. Thus, more work must be conducted to expand the knowledge in this area.

## Conclusion

The results of the item nonresponse analysis indicated the characteristics of patients with chronic disease in rural China. The respondents in the central provinces with low education levels, living with over eight household members, having multiple chronic diseases, and having low health knowledge levels tend to have more item nonresponse numbers, that is, they were more likely to keep silent. Interviewers should give more information and extend patience to respondents who inclined to keep silent. Meanwhile, the research indicated that the follow-up services in rural areas are insufficient. Policy-makers in the health field should pay attention to designing a supervision pattern of healthcare services and help health institutions enhance their service abilities. The study also showed that policy-makers should consider giving more support to vulnerable groups, such as patients with chronic disease or elderlies who live alone.
